# Late-onset schizophrenia patients with social phobia receive higher doses of antipsychotics compared patients without social phobia: results of an observational study

**DOI:** 10.1192/j.eurpsy.2022.641

**Published:** 2022-09-01

**Authors:** V. Pochueva, M. Savina, V. Sheshenin

**Affiliations:** FSBSI “Mental Health Research Centre”, Geriatric Psychiatry, Moscow, Russian Federation

**Keywords:** old age, late onset schizophrenia, antipsychotics, social phobia

## Abstract

**Introduction:**

Social phobia is frequent comorbidity in schizophrenia. It`s clinical correlates and consequencies for clinical practice in late-onset schizophrenia (LoS) are unclear.

**Objectives:**

The study aimed to compare clinical correlates and therapeutic options in LoS patient with and without social phobia.

**Methods:**

16 LoS patients with social phobia (ICD-11 diagnosis, age 59,6±6,2, 25% males) were compared with 16 LoS patients without social phobia (69,9±10,9, 0% males). Results of clinical assessment (PANSS, HDRS-17), cognitive examination (MMSE, MoCA), CT data were analysed. Type of antipsychotics (conventional\conconventional) was registed, dose of antipsychotics and antidepressants was randed from 1 (low) to 3 (high). Mann-Whitney test and χ^2^statistic was used.

**Results:**

There was no group differences in age, age manifestation, illness duration, number of psychotic episodes, PANSS and HADRS scores, rates results of cognitive tests, atrophy scores, length of hospital state, types of received antipsychotics. LoS patients with social phobia received more frequently medium and high doses of antipsychotics than LoS patient without social phobia (χ2 (2)=6,432, p=0,040).

**Conclusions:**

Increased doses of antypsychotics in patients with social phobia don`t correlate with severity of psychotic symptoms and nay reflect some treatment resistance as well as misinterpretation of symptoms of social phobia as insufficient retreat of the psychosis. Active detection of social phobia is significant for treatment optimization.

**Disclosure:**

No significant relationships.

